# A *major facilitator superfamily domain 8* frameshift variant in a cat with suspected neuronal ceroid lipofuscinosis

**DOI:** 10.1111/jvim.15663

**Published:** 2019-12-20

**Authors:** Julien Guevar, Petra Hug, Felix Giebels, Alexane Durand, Vidhya Jagannathan, Tosso Leeb

**Affiliations:** ^1^ Division of Clinical Neurology, Vetsuisse Faculty University of Bern Bern Switzerland; ^2^ Institute of Genetics, Vetsuisse Faculty University of Bern Bern Switzerland; ^3^ Division of Clinical Radiology, Vetsuisse Faculty University of Bern Bern Switzerland

**Keywords:** cat, genetics, lysosomal storage disease, MFSD8, NCL7, neuronal ceroid lipofuscinosis, precision medicine

## Abstract

A 2‐year‐old male domestic shorthair cat was presented for a progressive history of abnormal posture, behavior, and mentation. Menace response was absent bilaterally, and generalized tremors were identified on neurological examination. A neuroanatomical diagnosis of diffuse brain dysfunction was made. A neurodegenerative disorder was suspected. Magnetic resonance imaging findings further supported the clinical suspicion. Whole‐genome sequencing of the affected cat with filtering of variants against a database of unaffected cats was performed. Candidate variants were confirmed by Sanger sequencing followed by genotyping of a control population. Two homozygous private (unique to individual or families and therefore absent from the breed‐matched controlled population) protein‐changing variants in the *major facilitator superfamily domain 8* (*MFSD8*) gene, a known candidate gene for neuronal ceroid lipofuscinosis type 7 (CLN7), were identified. The affected cat was homozygous for the alternative allele at both variants. This is the first report of a pathogenic alteration of the *MFSD*8 gene in a cat strongly suspected to have CLN7.

**Abbreviations**
ADC: apparent diffusion coefficient
CCMD: macular dystrophy with central cone involvement
MFSD8: major facilitator superfamily domain 8
MRI: magnetic resonance imaging
MRS: magnetic resonance spectroscopy
NCBI: National Center for Biotechnology Information
NCL: neuronal ceroid lipofuscinosis
PCR: polymerase chain reaction
T2WI: T2‐weighted image

## 1 INTRODUCTION

The neuronal ceroid lipofucinoses (NCLs), also collectively called Batten disease, are a heterogeneous group of inherited, progressive, neurodegenerative disorders. In humans, 14 genetically distinct forms of NCL have been identified, and all are characterized by abnormal intralysosomal accumulation of autofluorescent material, which leads to progressive degeneration of cortical and cerebellar structures with secondary fiber tract atrophy. Each gene is called *CLN* (ceroid lipofuscinosis, neuronal) and given a different number designation as its subtype. The NCLs are among the most prevalent of the childhood neurodegenerative diseases.^1^ Despite genetic heterogeneity, the clinical phenotype often is similar and includes progressive loss of motor ability, cognitive deterioration, visual failure, and epileptic seizures.^2^ Although they are always fatal, death can occur early or later in life. The magnetic resonance imaging (MRI) features are cerebral and cerebellar atrophy, mild hyperintensity of the cerebral white matter on T2‐weighted images (T2WI), thinning of the cortex, and hypointensity of the thalami on T2WI, and their appearance correlates with the duration of the disease.^3^, ^4^ The NCLs feature an autosomal recessive mode of inheritance except CLN4, which has an autosomal dominant pattern.^1^ Different sequence variants can occur in a given gene and present with different clinical phenotypes. The CLN7 form has a late infantile onset and is caused by genetic variants in the *MFSD8* gene encoding a lysosomal protein termed major facilitator superfamily domain 8 (MFSD8). The MFSD8 protein is an atypical solute carrier protein whose physiological substrate is not known.^1^

The NCLs also have been reported in dogs but only rarely in cats.^5^, ^6^, ^7^, ^8^, ^9^, ^10^, ^11^, ^12^ So far, causative genetic variants in dogs are known for CLN1, 2, 5, 6, 7, 8, 10, and 12,^13^, ^14^, ^15^, ^16^, ^17^, ^18^, ^19^, ^20^, ^21^, ^22^ whereas none have been reported in cats. Clinically, for a dog to be considered a candidate for the disorder, progressive neurological signs including at least 4 of the following must be present: loss of vision, behavioral changes (eg, development of aggressive behavior), loss of learned behaviors, tremors, cerebellar ataxia, cognitive and motor decline, sleep disturbance, and seizures.^23^ The NCLs are invariably fatal, and the severity of the clinical signs often leads to euthanasia. Although not pathognomonic for the disease, the identification on MRI of cerebral and cerebellar atrophy with widened cerebral sulci, cerebellar folia, and increased volume in the ventricular system^23^, ^24^ further increases the antemortem suspicion. Similar clinical and imaging findings have been reported in cats.^5^, ^6^, ^7^, ^8^, ^9^, ^10^, ^11^, ^12^ Because the clinical signs and the imaging findings can overlap with other lysosomal storage diseases, identification of accumulation of autofluorescent ceroid storage material in neurological tissues is required for confirmation.^23^ Advances in genetics have permitted antemortem screening for genetic variants in known NCL candidate genes in suspected cases and have allowed for confirmation of NCL by demonstration that the disease results from a known NCL pathogenic sequence variant.^25^ In this report, we describe the clinical, imaging, and genetic findings in a cat with suspected NCL diagnosed antemortem.

## 2 MATERIALS AND METHODS

### 2.1 Animal selection/phenotyping

All animal experiments were performed according to local regulations. The cat in our study was privately owned and examined with the consent of the owner. The Cantonal Committee for Animal Experiments approved the collection of blood samples (Canton of Bern; permit 75/16).

**Figure 1:**
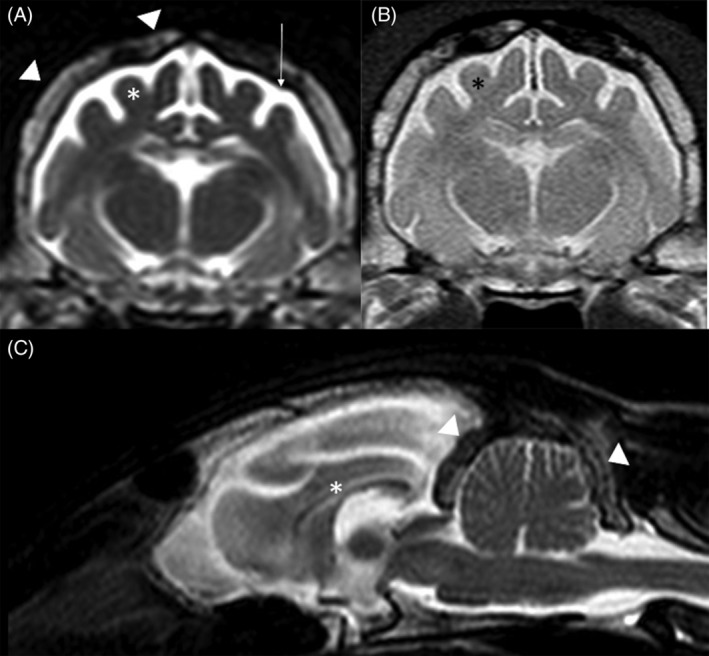
MRI of the brain in the affected cat. A, Transverse T2‐weighted image (T2WI) at the level of the thalamus. Diffuse cerebral atrophy (asterisk) with widened cerebral sulci and increased cerebrospinal fluid signal within the subarachnoid space (arrow). Marked hyperostosis is also identified (arrowhead). B, Transverse proton density‐weighted image at the same level than (A) highlights the poor demarcation between gray and white matter (asterisk). C, Midsagittal T2WI showing the diffuse increased signal intensity within the subarachnoid space secondary to the diffuse brain atrophy. Diffuse marked hyperostosis is also seen affecting the tentorium cerebelli and occipital bone (arrowhead). The corpus callosum is thin (asterisk), and there is mild widening of the cerebellar sulci

We selected 1 affected and 141 unaffected cats for inclusion in this study. The proband was a 2‐year‐old male domestic shorthair cat displaying aggression toward the owner and its housemate. It had always displayed an ataxic gait in all 4 limbs, but the gait disturbance became more evident around 6 months of age. A progressive crouched posture, loss of learned behavior, and abnormal mentation (disorientation) also were reported. Physical and neurological evaluation identified dull mentation, abnormal cranial nerves examination with absent menace bilaterally, normal pupillary light and dazzle reflexes, normal proprioception and segmental spinal reflexes in all 4 limbs. Generalized tremors also were observed. A neuroanatomic diagnosis of brain dysfunction with a diffuse disorder affecting the forebrain and cerebellum was made by a board‐certified veterinary neurologist. In light of the age of the cat and the progressive nature of the clinical signs, a neurodegenerative disorder was suspected. A CBC and serum biochemistry findings were normal, including microscopic assessment of the white blood cells. Urinalysis also was normal. Urine oligosaccharide and mucopolysaccharide concentrations testing were normal when compared to an age‐matched healthy control cat. Magnetic resonance imaging of the brain identified marked brain atrophy with decreased gray and white matter demarcation, thinning of the corpus callosum, mild widening of the cerebellar sulci, mild to moderate dilatation of the lateral and third ventricles, mild widening of the cerebellar sulci, and hyperostosis of the frontal, parietal, temporal, and occipital bones including the tentorium cerebelli (Figure 1). This imaging phenotype is similar than previously reported.^12^ Cerebrospinal fluid analysis was normal. Abdominal ultrasound examination was normal.

Unaffected control cats of various breeds were obtained from a pool of data from cats donated to the Vetsuisse Biobank. The relatedness among these cats was unknown. Genomic DNA was isolated from ethylenediaminetetraacetate blood with the Maxwell RSC Whole Blood Kit using a Maxwell RSC instrument (Promega).

### 2.2 Whole‐genome sequencing of an affected domestic shorthair cat

An Illumina TruSeq polymerase chain reaction (PCR)‐free DNA library with 350 base pair (bp) insert size was prepared from the affected cat (K548). We collected 286 million 2 × 150 bp paired‐end reads on a NovaSeq 6000 instrument (30 × coverage). Mapping and alignment were performed as previously described.^26^ The sequence data were deposited under study accession PRJEB7401 and sample accession SAMEA5885930 at the European Nucleotide Archive.

### 2.3 Variant calling

A variant filtering was performed.^26^ To predict the functional effects of the called variants, SnpEFF^27^ software together with National Center for Biotechnology Information (NCBI) annotation release 105 for felCat9 was used. For variant filtering, we used 38 control genomes, which were produced during other projects of our group.

### 2.4 Gene analysis

We used the *Felis catus* 9.0 reference genome assembly for all analyses. Numbering within the feline *MFSD8* gene corresponds to the NCBI RefSeq accessions XM_019828986.2 (mRNA) and XP_019684545.1 (protein).

### 2.5 Sanger sequencing

The *MFSD8*:c.19G>C variant and the *MFSD8*:c780delT were genotyped by direct Sanger sequencing of PCR amplicons. A 600 bp PCR product was amplified from genomic DNA using AmpliTaqGold360Mastermix (Life Technologies) together with primers 5′‐AGC CCT GTG TCT GTT CTG TG‐3′ (Primer F) and 5′‐AGG CAT ACG TTT TGT CAT GAT G‐3′ (Primer R) for the frameshift variant and primers 5′‐GAA AGC TAG GAC ACA GGG CC‐3′ (Primer F) and 5′‐CAC GTC TCC CAG AAG TTC CG‐3′ (Primer R) for the missense variant. After treatment with exonuclease I and alkaline phosphatase, amplicons were sequenced on an ABI 3730 DNA Analyzer (Life Technologies). Sanger sequences were analyzed using the Sequencher 5.1 software (GeneCodes).

## 3 RESULTS

### 3.1 Genetic analysis

We sequenced the genome of the affected cat and searched for homozygous and heterozygous variants that were not present in 38 control cats of different breeds (Table 1). We prioritized variants that were predicted to change the amino acid sequence of 1 of the known NCL candidate genes (Table S1).

**Table 1:** Results of variant filtering in the affected and 38 unaffected cats

Filtering step	Homozygous variants	Heterozygous variants
Private^a^ variants	45 586	109 336
Protein‐changing private variants	167	414
Private^a^ variants in known NCL candidate genes	2	0

a Private: Unique to individual or families and therefore absent from the breed‐matched controlled population.

We identified 2 homozygous Private (unique to individual or families and therefore absent from the breed‐matched controlled population) protein‐changing variants in known NCL candidate genes. Both variants were located in the *MFSD8* gene. A missense variant was located in the *MFSD8* gene and was designated ChrB1:98899302G>C (Felis_catus_9.0 assembly). This variant, XM_019828986.2:c.19G>C, is predicted to result in the amino acid change XP_019684545.1:p.(Asp7His). The second variant was a frameshift variant, also located in the *MFSD8* gene. It was designated as XM_019828986.2:c.780delT or XP_019684545.1:p.(Gln262Lysfs*33). The frameshift truncates 253 codons (49%) of the wild‐type sequence. The wild‐type MFSD8 protein consists of 514 amino acids including 12 transmembrane domains required for anchoring the mature protein in the lysosomal membrane. The mutant transcript lacks the coding information for the last 6 transmembrane domains. We confirmed the presence of both *MFSD8* variants by Sanger sequencing. The affected cat was homozygous for the alternative allele at both variants.

We also genotyped 141 control cats (Table S2). All control cats were homozygous for the wild‐type allele at both variants (Table 2) resulting in an allele frequency of 0%.

**Table 2:** Genotype phenotype association of the *MFSD8*:c.19G>C and c.780delT variants

	c.19G>C		c.780delT	
	G/G	C/C	T/T	del/del
Case	…	1	…	1
Controls	141	…	141	…

## 4 DISCUSSION

We identified a missense and a frameshift variant in the *MFSD8* gene in a cat with clinical and imaging phenotype highly compatible with NCL. The clinical, imaging, and genetic findings together strongly support the diagnosis of CLN7.

The clinical features of 8 cats with NCL confirmed by histopathology have been reported since 1974.^5^, ^6^, ^7^, ^8^, ^9^, ^10^, ^11^, ^12^ Domestic shorthair (7/9) cats and Siamese (2/9) are the 2 reported breeds. Most cats were young adults by the time they were assessed (1‐2 years old) although earlier clinical signs were reported at a few months of age. In 9 cats (including ours), the most commonly reported clinical signs were vision loss (6/9), seizures (5/9), abnormal mentation (4/9), abnormal behavior (4/9), abnormal gait (4/9), hyperesthesia (3/9), tremors (3/9), and myoclonus (2/9). Evidence of progressive forebrain signs in a young adult cat therefore should raise the suspicion for NCL.

The MRI characteristics of 1 cat with confirmed NCL have been reported.^12^ The findings included generalized and symmetrical brain cortical atrophy with secondary dilatation of the ventricular system and intracranial subarachnoid space as observed in our cat. Reduction in size of the corpus callosum also was reported. These findings are similar to those in affected dogs and humans.^3^, ^4^, ^23^ Furthermore, in humans, longitudinal MRI and magnetic resonance spectroscopy (MRS) imaging have identified cortical gray matter volumes that decreased substantially over time with proton MRS reflecting this finding with decreasing *N*‐acetyl aspartate (neuronal marker) and increasing myoinositol (gliosis marker). Diffusion weighted imaging also has been evaluated to supplement the clinical disability scale in an attempt to provide a quantitative assessment of neurodegeneration. Although higher apparent diffusion coefficients (ADC) were found in NCL‐affected children compared to age‐matched controls, correlation with the degree of disability was not statistically significant. The role of MRS and ADC in cats with NCL is unknown. The general calvarial hyperostosis identified in both cats (our cat and the previously reported cat^12^) is suspected to be a dynamic relationship in brain size and skull thickness.^28^

Both detected protein‐changing variants in the *MFSD8* gene were in perfect linkage disequilibrium. Based on the available data, it therefore is impossible to make conclusive statements about the functional relevance of the individual variants. The p.Asp7His missense variant might be a functionally neutral variant that merely is in strong linkage disequilibrium with the true causative variant. The c.780delT variant causes a frameshift and thus most likely leads to a complete loss of function of the *MFSD8* gene. Given the known impact of *MFSD8* variants in other species, it therefore seems highly probable that this variant is pathogenic and responsible for the NCL phenotype in the affected cat. The genetic data strongly suggest that this case should be classified as CLN7 because it is most likely due to a deficiency in MFSD8.

In humans, 30 different genetic variants have been identified in *CLN*7 comprising missense, nonsense, or deletion/frameshift variants affecting protein function.^29^ Across species, genetic rodent models of CLN7 have been created and spontaneous large animal models of CLN7 have been reported in dogs (Chinese crested dogs^19^ and Chihuahuas^18^, ^22^) and non‐human primates (Japanese macaques^29^). When comparing neurological findings of our cat and the reported NCL cats with the 2 aforementioned species, head tilt (dog and macaque) and hypermetria (macaque) were never a feature in any of the cats. Genetic variants of *MFSD8* also were reported in human patients with ocular disease (nonsyndromic macular dystrophy with central cone involvement [CCMD]) without neurological features, suggesting that NCL and CCMD are likely not different disease entities but rather allelic diseases.^30^

The main limitations of our study are the examination of a single affected cat and the absence of histopathology. The established importance of the *MFSD*8 gene as a cause of CLN7 in several other species, the predicted effect of the variant, and the absence of the mutant allele in the control cats all suggest that the frameshift variant is pathogenic and the cause of the observed NCL in this cat.

## CONFLICT OF INTEREST DECLARATION

Authors declare no conflict of interest.

## OFF‐LABEL ANTIMICROBIAL DECLARATION

Authors declare no off‐label use of antimicrobials.

## INSTITUTIONAL ANIMAL CARE AND USE COMMITTEE (IACUC) OR OTHER APPROVAL DECLARATION

All animal experiments were performed according to local regulations. The cat in this study is privately owned and was examined with the consent of the owner. The Cantonal Committee for Animal Experiments approved the collection of blood samples (Canton of Bern; permit 75/16).

## HUMAN ETHICS APPROVAL DECLARATION

Authors declare human ethics approval was not needed for this study.

## Supporting information


**Table S1** Known genes for neuronal ceroid lipofuscinoses in human and veterinary medicine.

**Table S2** Genotypes of cats at the MFSD8 protein‐changing variants. The NCL affected cat is indicated in red.
