# Supplementary material for: A major facilitator superfamily domain 8 frameshift variant in a cat with suspected neuronal ceroid lipofuscinosis
Source: J Vet Intern Med. 2019 Dec 20;34(1):289–93. doi: 10.1111/jvim.15663 (PMC6979099; doi:10.1111/jvim.15663)
Supplement: Supplementary file 1 — Table S1 Known genes for neuronal ceroid lipofuscinoses in human and veterinary medicine. [file JVIM-34-289-s001.pdf]

Table S1. Known genes for neuronal ceroid lipofuscinoses in human and veterinary medicine.

Human disease designation	Phenotype MIM number	Inheritance	Gene	Remark	Online Mendelian Inheritance in Animals (OMIA)	Species
CLN1	256730	AR	*PPT1*		001504-9615	dog
CLN2	204500	AR	*TPP1*		001472-9615	dog
CLN3	204200	AR	*CLN3*			
CLN4A	204300	AR	*CLN6*	allelic to CLN6		
CLN4B	162350	AD	*DNAJC5*			
CLN5	256731	AR	*CLN5*		001482-9615, 001482-9913, 001482-9940	dog, cattle, sheep
CLN6	601780	AR	*CLN6*		001443-9615, 001443-9940	dog, sheep
CLN7	610951	AR	*MFSD8*		001962-9615, 001962-9542	dog, *Macaca fusacata*
CLN8	600143	AR	*CLN8*		001506-9615	dog
CLN9	609055	AR	unknown			
CLN10	610127	AR	*CTSD*		001505-9615	dog, sheep
CLN11	614706	AR	*GRN*			
"CLN12", Kufor-Rakeb syndrome	606693	AR	*ATP13A2*		001552-9615	dog
CLN13	615362	AR	*CTSF*			
"CLN14", epilepsy, progressive myoclonic 3, with or without intracellular inclusions	611726	AR	*KCTD7*			
