# Supplementary material for: A major facilitator superfamily domain 8 frameshift variant in a cat with suspected neuronal ceroid lipofuscinosis
Source: J Vet Intern Med. 2019 Dec 20;34(1):289–93. doi: 10.1111/jvim.15663 (PMC6979099; doi:10.1111/jvim.15663)
Supplement: Supplementary file 2 — Table S2 Genotypes of cats at the MFSD8 protein‐changing variants. The NCL affected cat is indicated in red. [file JVIM-34-289-s002.pdf]

Table S2. Genotypes of cats at the MFSD8 protein-changing variants. The NCL affected cat is indicated in red.

Lab Id	Breed	Phenotype	MFSD8:c.19G>C	MFSD8:c.780delT
K003	European Shorthair	control	G/G	T/T
K004	European Shorthair	control	G/G	T/T
K006	European Shorthair	control	G/G	T/T
K007	European Shorthair	control	G/G	T/T
K010	European Shorthair	control	G/G	T/T
K013	European Shorthair	control	G/G	T/T
K026	European Shorthair	control	G/G	T/T
K027	European Shorthair	control	G/G	T/T
K028	Ragdoll	control	G/G	T/T
K029	European Shorthair	control	G/G	T/T
K032	European Shorthair	control	G/G	T/T
K044	European Shorthair	control	G/G	T/T
K046	European Shorthair	control	G/G	T/T
K051	European Shorthair	control	G/G	T/T
K062	European Shorthair	control	G/G	T/T
K066	European Shorthair	control	G/G	T/T
K078	unknown	control	G/G	T/T
K079	unknown	control	G/G	T/T
K081	European Shorthair	control	G/G	T/T
K084	European Shorthair	control	G/G	T/T
K087	European Shorthair	control	G/G	T/T
K088	European Shorthair	control	G/G	T/T
K089	European Shorthair	control	G/G	T/T
K091	European Shorthair	control	G/G	T/T
K093	European Shorthair	control	G/G	T/T
K094	European Shorthair	control	G/G	T/T
K099	European Shorthair	control	G/G	T/T
K100	European Shorthair	control	G/G	T/T
K101	unknown	control	G/G	T/T
K104	Sacred Cat of Burma	control	G/G	T/T
K106	Maine Coon	control	G/G	T/T
K108	unknown	control	G/G	T/T
K109	Maine Coon	control	G/G	T/T
K110	Maine Coon	control	G/G	T/T
K113	Maine Coon	control	G/G	T/T
K119	Maine Coon	control	G/G	T/T
K120	Maine Coon	control	G/G	T/T
K121	Maine Coon	control	G/G	T/T
K125	Maine Coon	control	G/G	T/T
K161	Maine Coon	control	G/G	T/T
K168	Maine Coon	control	G/G	T/T
K180	Maine Coon	control	G/G	T/T
K187	Maine Coon	control	G/G	T/T

K192	Maine Coon	control	G/G	T/T
K203	Maine Coon	control	G/G	T/T
K216	Maine Coon	control	G/G	T/T
K221	Maine Coon	control	G/G	T/T
K222	Maine Coon	control	G/G	T/T
K223	Maine Coon	control	G/G	T/T
K224	Maine Coon	control	G/G	T/T
K227	Maine Coon	control	G/G	T/T
K228	Maine Coon	control	G/G	T/T
K229	Maine Coon	control	G/G	T/T
K230	Maine Coon	control	G/G	T/T
K231	Maine Coon	control	G/G	T/T
K232	Maine Coon	control	G/G	T/T
K233	Maine Coon	control	G/G	T/T
K234	Maine Coon	control	G/G	T/T
K235	Maine Coon	control	G/G	T/T
K236	Maine Coon	control	G/G	T/T
K237	Maine Coon	control	G/G	T/T
K238	Maine Coon	control	G/G	T/T
K239	Maine Coon	control	G/G	T/T
K240	Maine Coon	control	G/G	T/T
K241	Maine Coon	control	G/G	T/T
K243	Maine Coon	control	G/G	T/T
K245	Maine Coon	control	G/G	T/T
K246	Maine Coon	control	G/G	T/T
K247	Maine Coon	control	G/G	T/T
K248	Maine Coon	control	G/G	T/T
K249	Maine Coon	control	G/G	T/T
K250	Maine Coon	control	G/G	T/T
K251	Maine Coon	control	G/G	T/T
K252	Maine Coon	control	G/G	T/T
K253	Maine Coon	control	G/G	T/T
K254	Maine Coon	control	G/G	T/T
K255	Maine Coon	control	G/G	T/T
K256	Maine Coon	control	G/G	T/T
K257	Maine Coon	control	G/G	T/T
K258	Maine Coon	control	G/G	T/T
K259	Maine Coon	control	G/G	T/T
K260	Maine Coon	control	G/G	T/T
K313	Maine Coon	control	G/G	T/T
K383	Maine Coon	control	G/G	T/T
K389	Maine Coon	control	G/G	T/T
K400	Maine Coon	control	G/G	T/T
K403	Maine Coon	control	G/G	T/T
K410	Maine Coon	control	G/G	T/T
K413	Maine Coon	control	G/G	T/T
K414	Maine Coon	control	G/G	T/T

K427	Maine Coon	control	G/G	T/T
K428	Maine Coon	control	G/G	T/T
K429	Maine Coon	control	G/G	T/T
K430	Maine Coon	control	G/G	T/T
K438	Sacred Cat of Burma	control	G/G	T/T
K440	unknown	control	G/G	T/T
K445	unknown	control	G/G	T/T
K446	unknown	control	G/G	T/T
K449	Don Sphynx	control	G/G	T/T
K453	Sacred Cat of Burma	control	G/G	T/T
K454	Sacred Cat of Burma	control	G/G	T/T
K459	unknown	control	G/G	T/T
K460	Somali	control	G/G	T/T
K461	Somali	control	G/G	T/T
K462	Somali	control	G/G	T/T
K463	Somali	control	G/G	T/T
K464	Somali	control	G/G	T/T
K465	Egyptian Mau	control	G/G	T/T
K466	Egyptian Mau	control	G/G	T/T
K468	Egyptian Mau	control	G/G	T/T
K470	Egyptian Mau	control	G/G	T/T
K476	Egyptian Mau	control	G/G	T/T
K477	Egyptian Mau	control	G/G	T/T
K478	Egyptian Mau	control	G/G	T/T
K480	Egyptian Mau	control	G/G	T/T
K481	Egyptian Mau	control	G/G	T/T
K483	Egyptian Mau	control	G/G	T/T
K487	Egyptian Mau	control	G/G	T/T
K489	Maine Coon	control	G/G	T/T
K490	Maine Coon	control	G/G	T/T
K491	Maine Coon	control	G/G	T/T
K492	Maine Coon	control	G/G	T/T
K493	Maine Coon	control	G/G	T/T
K497	Egyptian Mau	control	G/G	T/T
K498	Sphnx	control	G/G	T/T
K501	Norwegian Forest cat	control	G/G	T/T
K503	Burmese	control	G/G	T/T
K504	Abessinier	control	G/G	T/T
K505	Abessinier	control	G/G	T/T
K506	Abessinier	control	G/G	T/T
K507	Somali	control	G/G	T/T
K508	Abessinier	control	G/G	T/T
K512	unknown	control	G/G	T/T
K516	Maine Coon	control	G/G	T/T
K521	Burmese	control	G/G	T/T
K534	Domestic Shorthair	control	G/G	T/T
K545	European Longhair	control	G/G	T/T

K547	Bengal	control	G/G	T/T
**K548**	**European Shorthair**	**NCL affected**	**C/C**	**del/del**
K550	European Shorthair	control	G/G	T/T
K551	European Shorthair	control	G/G	T/T
K557	Peterbald	control	G/G	T/T
